# Supplementary material for: FERN – a Java framework for stochastic simulation and evaluation of reaction networks
Source: BMC Bioinformatics. 2008 Aug 29;9:356. doi: 10.1186/1471-2105-9-356 (PMC2553347; doi:10.1186/1471-2105-9-356)
Supplement: Additional file 1 — FERN distribution, Version 1.3. This archive contains the FERN source code and binaries as well as documentation and example models in FernML and SBML. [file 1471-2105-9-356-S1.zip › fern/doc/javadoc/index-files/index-2.html]

B-Index


---


|  |  |  |
| --- | --- | --- |
| **Overview** \| Package \| Class \| Use \| **Tree** \| **Deprecated** \| **Index** \| **Help** | |  |
| **PREV LETTER**   **NEXT LETTER** | **FRAMES**    **NO FRAMES**     **All Classes** |  |


A B C D E F G H I J L M N O P R S T U V 

---


## **B**

**Benchmark** - Class in fern.benchmark: Base class for all benchmark classes. **Benchmark()** - Constructor for class fern.benchmark.Benchmark: **benchmark()** - Method in class fern.benchmark.SimulatorPerformance: Performs one benchmark for each of the six simulators. **benchmarkLangevinAgainstTauLeapNumberGeneration(double[])** - Method in class fern.benchmark.RandomNumber: Benchmark for the different computation of random numbers in the Langevin (normal distributed) and tau leap (poisson distributed) method for double values at **bfs(int[], int[], NetworkSearchAction)** - Method in class fern.analysis.AnalysisBase: Performs a breath first search starting at the given sources (which means the contents of `speciesSource` and `reactionSource` are the initial content of the queue.

---


|  |  |  |
| --- | --- | --- |
| **Overview** \| Package \| Class \| Use \| **Tree** \| **Deprecated** \| **Index** \| **Help** | |  |
| **PREV LETTER**   **NEXT LETTER** | **FRAMES**    **NO FRAMES**     **All Classes** |  |


A B C D E F G H I J L M N O P R S T U V 

---
